# Intestinal and hepatic benefits of BBR-EVO on DSS-induced experimental colitis in mice

**DOI:** 10.3389/fmicb.2024.1428327

**Published:** 2024-09-04

**Authors:** Wenjia Wang, Yiheng Han, Wen Yin, Qiaozhi Wang, Yi Wu, Maobo Du

**Affiliations:** ^1^Institute of Chinese Materia Medica, China Academy of Chinese Medical Sciences, Beijing, China; ^2^College of Animal Science, Ningxing University, Yinchuan, China; ^3^College of Veterinary Medicine, Nanjing Agricultural University, Nanjing, China; ^4^Changzhou Hospital Affiliated to Nanjing University of Chinese Medicine, Changzhou, China; ^5^College of Veterinary Medicine, Yunnan Agricultural University, Kunming, China

**Keywords:** Yulian decoction, BBR-EVO, DSS colitis, liver, intestinal dysbiosis

## Abstract

Ulcerative colitis (UC), characterized by disrupted intestinal barrier integrity and chronic inflammation, was modeled in mice via dextran sulfate sodium (DSS) induction. This study explored the therapeutic potential of berberine-evodiamine (BBR-EVO), bioactive components of the traditional Chinese medicine Yulian decoction, in DSS colitis. BBR-EVO intervention ameliorated weight loss, diarrhea, colonic shortening, and histopathological damage in colitic mice. The substance increased antioxidant activity while reducing high levels of pro-inflammatory cytokines in the colon, including as TNF-α, IL-1β, and IL-6. BBR-EVO inhibited the DSS-induced decrease in the tight junction proteins ZO-1 and occludin, according to immunohistochemistry. 16S rRNA sequencing demonstrated BBR-EVO partially attenuated DSS-elicited intestinal dysbiosis, reducing opportunistic pathogens and restoring diminished beneficial taxa. Critically, BBR-EVO alleviated secondary hepatic injury in colitic mice, mitigating immune cell infiltration, oxidative stress, cytokine production, and ultrastructural damage, likely by beneficially modulating gut-liver crosstalk. This study reveals BBR-EVO, derived from a traditional Chinese medicine, confers multi-target protective effects in experimental colitis and associated hepatic pathology, warranting further evaluation as a potential therapy for inflammatory bowel diseases like UC. The mechanisms may involve simultaneous augmentation of intestinal barrier integrity, inhibition of inflammation, microbiota regulation, and gut-liver axis optimization.

## Introduction

Ulcerative colitis (UC) represents a common subtype of inflammatory bowel disease (IBD) affecting the human population. The clinical manifestations of UC encompass anorexia, abdominal discomfort, hematochezia, and mucosal discharge (Eom et al., 2018). Statistical evidence suggests that IBD impacts an estimated 50,000 people worldwide, with an ascending prevalence (Le Berre et al., 2023). In Western nations, the prevalence of IBD might surpass 0.5% (Kaplan, 2015; Du and Ha, 2020). Nonetheless, the etiology of UC remains elusive, and an absolute cure has not been established. Research has demonstrated that both genetic predispositions and environmental influences elevate the risk for UC (Cosnes et al., 2011). Furthermore, dysregulation of immune responses, disruptions in intestinal microbiota, and other contributory factors are associated with UC (Kaser et al., 2010). Substantial evidence indicates that the human gut microbiota consists of a diverse array of symbiotic strains and microorganisms (Jia et al., 2008), with significant alterations observed in the composition and function of this microbiota in IBD patients (Lavelle and Sokol, 2020). Patients with IBD present with dysbiosis of gut microbiota, resulting in compromised intestinal epithelial barrier integrity, heightened intestinal permeability, and the ensuing translocation of endotoxins (e.g., lipopolysaccharides, LPS) into the circulatory system, which provokes an inflammatory response in the gut (Ungaro et al., 2017; Zhang et al., 2017; Du and Ha, 2020), This sequence of events can precipitate the activation of the hepatic immune system, potentially culminating in liver damage (Fukui, 2015). Investigations have identified oxidative stress, dysbiosis of the intestinal microbiota, and inflammation as shared pathogenic elements contributing to both liver injury and IBD (Chao et al., 2016; Rogler et al., 2021). Considering the liver’s heightened exposure to bacterial toxins, the dysbiosis observed in colitis may be implicated in hepatic injury via the gut-liver axis. Damage to the intestinal barrier permits the translocation of bacterial metabolites and toxins to the liver, which may induce a spectrum of hepatic diseases (Ohtani and Kawada, 2019).

The persistence of a high recurrence risk in patients with inflammatory bowel disease (IBD) post-medication necessitates further investigation into potent adjuvant therapies. With its therapeutic approach based on the principles of holistic and balanced care, Traditional Chinese Medicine (TCM) is becoming more and more acknowledged as a valuable complementary and alternative modality. It offers sustained therapeutic outcomes, minimal side effects, and a diverse range of bioactive constituents with multi-target effects (Kaplan, 2015). Given its oral administration, TCM primarily acts through the gastrointestinal system, a key site for absorption and metabolic processing. Notably, Traditional Chinese medicine (TCM) has been extensively utilized for managing ulcerative colitis, owing to its overall efficacy in restoring intestinal flora balance (Chen et al., 2015), along with its antidiarrheal and anti-inflammatory properties. Yulian decoction comprises coptidis, evodia fructus, and acostalis, and has purgative and analgesic effects. It is commonly prescribed for epigastric pain, sour belching, and diarrhea. Modern pharmacology has validated the analgesic, anti-ulcer, and antibacterial properties of Yulian decoction (Wang L. et al., 2011; Wang Y. et al., 2011). *In vitro* studies have confirmed the anti-inflammatory activity of compounds in Yulian decoction, which can suppress TNF-α and IL-6 levels in inflammatory cell models (Ran et al., 2022). Moreover, recent studies have identified 11 bioactive compounds in Yulian decoction, including berberine (BBR) and evodiamine (EVO), present in rat plasma (Li T. et al., 2021), while some *in vivo* studies indicated the protective effect of berberine on acute gastric ulcer (Guo et al., 2024). Investigating the synergistic effects of two active compounds, BBR and EVO, in Yulian decoction on the intestinal milieu may prove pivotal in demystifying the underlying pharmacological mechanisms.

## Materials and methods

### Animals

Male ICR mice, with an average weight of 25 ± 2 g, were acquired from the Comparative Medicine Centre at Yangzhou University (Yangzhou, China). These animals were maintained in a controlled environment, with a temperature of 22 ± 2°C, relative humidity of 50 ± 10%, and a 12-h light–dark cycle, with *ad libitum* access to food and water.

### Chemicals and reagents

**Figure 1:**
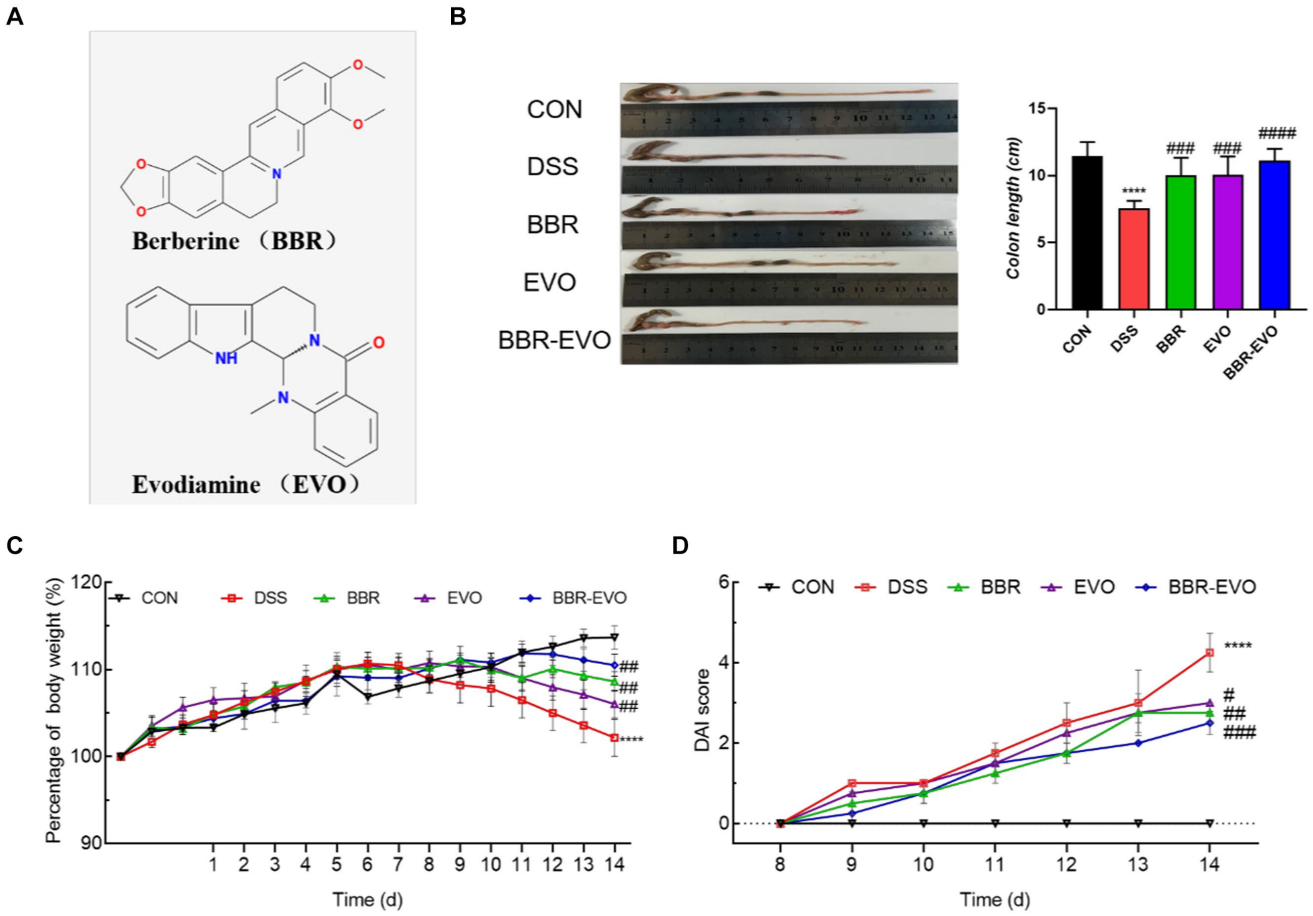
The main active components of Yulian decoction treatment relieve DSS-induced colitis. **(A)** Chemical structures of berberine and evodiamine. **(B)** The results of mice colon length, *n* = 8. **(C)** Variations in mice body weight. **(D)** Disease Activity Index (DAI) scores during the colitis induction period. Data are presented by mean ± SD; ^*^p < 0.05, ^**^p < 0.01, ^***^p < 0.001, ^****^p < 0.0001, compared to CON group; ^#^*p* < 0.05, ^##^*p* < 0.01, ^###^*p* < 0.001, ^####^*p* < 0.0001, compared to DSS group.

Berberine (BBR, IB0440, Beijing) and evodiamine (EVO, IE0430) were purchased from Solarbio (Beijing, China). BBR and EVO are initially dissolved in DMSO to prepare 100 mM stock solutions (Figure 1A). Dextran Sulfate Sodium Salt (DSS, 60316ES60) was provided from Yeasen (Shanghai, China).

### Animal experimental design

Following a one-week acclimatization period, all mice were randomly allocated into five distinct groups (n = 8 per group): control (CON), dextran sulfate sodium saline (DSS), berberine (BBR), evodiamine (EVO), and combination of BBR and EVO (BBR-EVO) group. The BBR and EVO content in the lyophilized Yulian decoction were 9.14 and 0.065%, respectively (Li T. et al., 2021). The ratio of BBR to EVO was maintained to reflect their proportions in the original Yulian decoction, aiming to preserve potential synergistic effects while ensuring physiological relevance. Dose extrapolation from a 70 kg human adult to rats (0.05 g/kg) was performed using a body surface area normalization factor of 0.018 (Xu, 1991), and an analogous calculation was employed for mice using a factor of 0.0026. Consequently, the resultant mice equivalent dose was 0.072 g/kg, with a daily administration of BBR at 19.74 mg/kg and EVO at 0.141 mg/kg.

Subsequent to the acclimatization phase, mice in the pharmacologically treated cohorts were given oral BBR, EVO, or BBR-EVO. In contrast, mice in the CON and DSS cohorts received daily PBS for 14 days. Commencing on the 7th day of treatment, all groups except for the CON group were provided with 3% (w/v) DSS in their drinking water for seven consecutive days, with daily renewal of the DSS solution, to induce a mouse model of acute ulcerative colitis. The assessment of disease severity in murine models was conducted through the application of the Disease Activity Index (DAI) scoring system. This index encompasses a comprehensive evaluation of physiological parameters, namely fecal consistency and bleeding (Camuesco et al., 2004). Each parameter was meticulously observed and assigned a score based on established diagnostic guidelines.

### Inflammatory cytokine analysis and antioxidant capacity analyses by ELISA

For preparation of tissue homogenates, samples from either the colon or liver were meticulously weighed and homogenized with pre-chilled normal saline in a ratio of 1:9 (m/v). The homogenization process was carried out at a temperature of 4°C. Subsequently, the homogenates were centrifuged at 12,000 rpm for 10 min, and the supernatant was collected. The concentrations of Total Antioxidant Capacity (T-AOC, YH1246), Malondialdehyde (MDA, YH1217), Superoxide Dismutase (SOD, YH1202), and Interleukin-6 (IL-6, ANG-E21044M), −1β (IL-1β, ANG-E21027M), and tumor necrosis factor -а (TNF-а, ANG-E21030M) were quantified using appropriate commercial ELISA kits, purchased from Nanjing Angle gene Biotechnology Co., Ltd. (China).

### Histopathological analysis

In line with the methodology outlined by Wang et al. (2019), hematoxylin and eosin (H&E) staining was employed to evaluate the histopathological alterations in the mouse colon and liver. Tissue samples fixed in 4% paraformaldehyde were sequentially dehydrated using graded ethanol solutions prior to a three-hour wax immersion. Subsequently, mouse colon and liver samples were sectioned to a thickness of 5 micrometers (μm). These sections were subsequently rehydrated, cleared of wax using xylene, and stained with hematoxylin and eosin, following standard histological protocols. Finally, morphological changes in the tissues were assessed and documented using an Olympus optical microscope (Olympus, Japan) and a Leica microscopic imaging system (Leica Biosystems, Germany).

### Immunohistochemistry analysis

Briefly, for antigen retrieval, the paraffin-embedded slides were submerged in 0.01 mM citrate buffer and microwaved for 10 min. Following a phosphate-buffered saline (PBS) wash, the slides were incubated at room temperature, first with 3% hydrogen peroxide (H₂O₂) for 10 min and then with goat serum for 15 min. The slides were then treated with goat anti-rabbit secondary antibody, followed by a 30-min incubation at room temperature. Subsequent to a PBS rinse, each slide was covered with a diaminobenzidine (DAB) solution (DA1010, Solarbio, China) and allowed to react at room temperature. One minute later, each slide was gently washed with water, counterstained with hematoxylin, dehydrated, and sealed with neutral gum before storage for subsequent analysis. Microscopic images of the stained sections were captured using a Nikon optical microscope (Nikon, Japan).

### Western blot analysis

Approximately 50 mg of colon tissue was mixed with an appropriate volume of pre-cooled PIPA Lysis Buffer and then completely lysed using a low-temperature homogenizer (Servicebio, Wuhan, China) for 10 to 15 min. The supernatant was then collected following centrifugation at 12,000 rpm for 20 min. Upon determining the protein concentration, samples were normalized to a uniform concentration and subjected to denaturation. Resolving gels of varying concentrations were prepared in accordance with the molecular weight of the target protein. The protein samples were then subjected to electrophoresis, followed by transfer onto a polyvinylidene difluoride (PVDF) membrane. The PVDF membrane was blocked using a Tris-buffered saline with Tween (TBST) containing 5% skimmed milk, followed by incubation with the primary antibody diluent overnight at 4°C. Following washes with TBST, the membrane was incubated with the secondary antibody diluent for 1.5 h at room temperature. Details of the antibodies utilized are listed in Table 1. Subsequently, the membranes were treated with a chemiluminescent HRP substrate solution (Biosharp, Anhui, China). Signals on the PVDF membranes were detected using a Bio-Rad chemiluminescence imaging system. Band densities were quantitatively analyzed using ImageJ software, with all data normalized to β-actin levels prior to analysis.

**Table 1:** F/B values in each group mice.

Group	Firmicutes (%)	Bacteroidetes (%)	F/B
CON	52.25	31.92	1.64
DSS	19.07	11.07	1.72
BBR-EVO	38.91	24.07	1.62

### Extraction of microbial genomic DNA from murine intestinal content

Genomic DNA was extracted from the intestinal content samples of mice belonging to three experimental cohorts (designated as BBR, EVO, and BBR-EVO group) employing the QIAamp DNA Mini Kit (QIAGEN, Germany). The quantification of genomic DNA was achieved through spectrophotometric analysis using the NanoDrop technology (Thermo Scientific, Wilmington), while the DNA integrity was ascertained by conducting electrophoresis on a 1% agarose gel at 150 V for a duration of 40 min. Purified DNA samples were stored at −80°C pending further analytical procedures.

### Amplification and sequencing of bacterial 16 s rRNA genes

The amplification of the V3-V4 hypervariable regions of the bacterial 16S rRNA genes was conducted using the primer pair 338F (5′-ACTCCTACGGGAGGCAGCA-3′) and 806R (5′-GGACTACHVGGGTWTCTAAT-3′). The resultant PCR products were then subjected to a purification process employing Vazyme VAHTSTM DNA Clean Beads (Vazyme, China), with an ensuing validation step via 1% agarose gel electrophoresis. Sequencing libraries were meticulously prepared from the purified amplicons to ensure a singular peak at a standard concentration of 2 nM. High-throughput sequencing was executed on an Illumina HiSeq platform (Illumina, San Diego, USA), facilitating a comprehensive analysis of the bacterial genomic composition. Experimental results are uploaded to the Genescloud Platform for microbiome bioinformatics analysis.^1^

### Statistical analysis

Data are expressed as the mean ± standard deviation (SD). Differences between groups were analyzed using the one-way ANOVA test followed by the Tukey test for multiple comparisons for normally distributed data, or the Kruskal-Wallis test followed by Dunn’s test for multiple comparisons for non-normally distributed data. A *p*-value ≤0.05 was deemed to denote statistical significance.

## Result

### BBR-EVO ameliorated DSS-induced colitis in mice

Mice in the DSS group exhibited a reduction in body weight and a decrease in colon length compared to the CON group (Figures 1B, C). In contrast, mice treated with BBR, EVO, and BBR-EVO showed an increase in both body weight and colon length relative to the DSS group. Notably, the BBR-EVO combination was more effective in improving DAI scores (Figure 1D).

**Figure 2:**
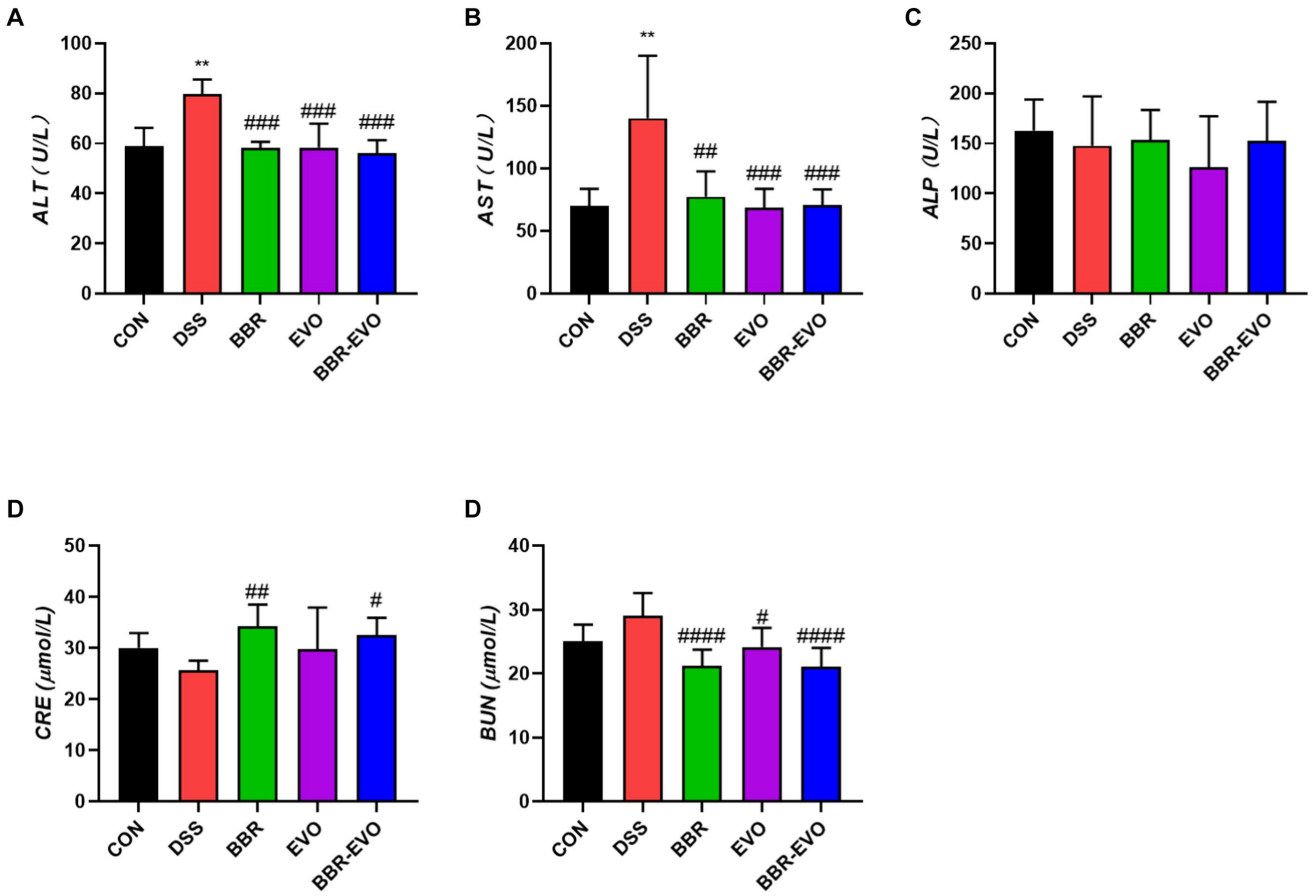
Effects of main active components of Yulian Decoction treatment on blood biochemical indices in mice, *n* = 5. **(A)** Alanine aminotransferase (ALT). **(B)** Aspartate aminotransferase (AST). **(C)** Alkaline phosphatase (ALP). **(D)** Creatinine (CRE). **(E)** Blood urea nitrogen (BUN). Data are presented by mean ± SD; ^*^p < 0.05, ^**^p < 0.01, ^***^p < 0.001, ^****^p < 0.0001, compared to CON group; ^#^*p* < 0.05, ^##^*p* < 0.01, ^###^*p* < 0.001, ^####^*p* < 0.0001, compared to DSS group.

As demonstrated in Figure 2, as compared to the CON group, the levels of ALT and AST in the DSS group’s serum increased considerably (*p* < 0.01), whereas the levels of ALP, BUN, and CRE kept unchanged. After BBR and BBR-EVO interventions, ALT and AST levels reduced dramatically (*p* < 0.001), CRE levels increased significantly (*p* < 0.05), and BUN levels increased significantly (*p* < 0.0001). BUN levels rose considerably during EVO intervention (*p* < 0.05).

**Figure 3:**
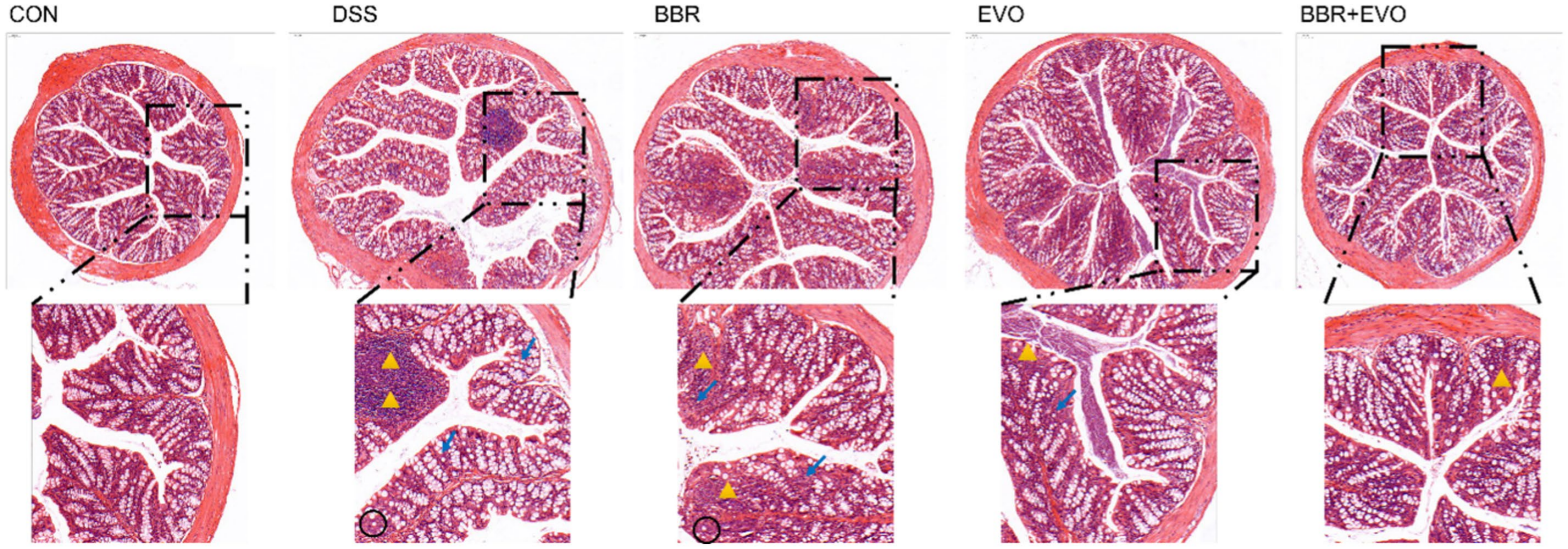
Effects of the main active components of Yulian decoction on histopathological changes in the mouse colon assessed by hematoxylin and eosin staining (H&E). Scale bar = 100 μm. Black circles indicate decreased goblet cells; yellow triangles indicate inflammatory infiltrate; blue arrows indicate abnormal crypt structure.

Hematoxylin and Eosin (HE) staining facilitated the histological examination of colon tissue, as depicted in Figure 3. The CON group exhibited a normal colonic mucosal structure. In stark contrast, the DSS group’s colon tissue displayed significant disruption of colonic crypts, abnormal gland distribution, and elevated inflammatory cell infiltration. Following pretreatment with BBR, EVO, and particularly the BBR-EVO combination, there was notable tissue repair. The colon tissues exhibited intact crypts and reduced inflammation. Most remarkably, the BBR-EVO group’s colon tissue closely resembled that of the CON group, with a successfully restored structure and slightly inflammation.

### BBR-EVO alleviated the inflammatory response of colon tissue in DSS-induced colitis mice

**Figure 4:**
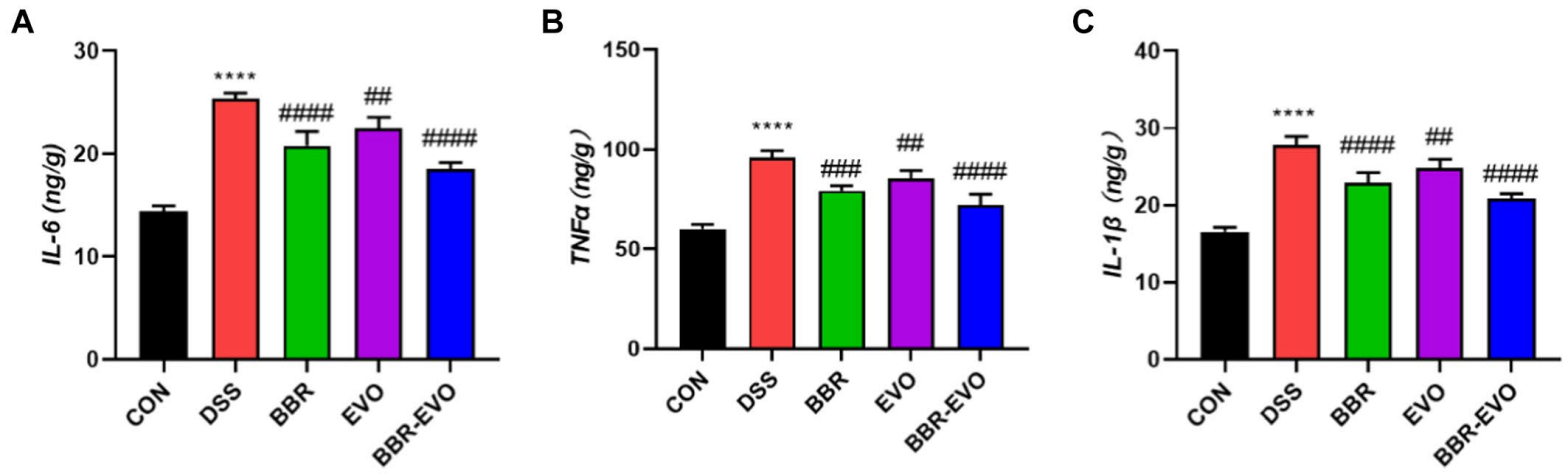
Levels of pro-inflammatory cytokines in mouse colon tissues as determined by enzyme-linked immunosorbent assay (ELISA), *n* = 5. **(A)** Interleukin-6 (IL-6). **(B)** Tumor necrosis factor alpha (TNF-α). **(C)** Interleukin-1 beta (IL-1β). Data are presented by mean ± SD; ^*^p < 0.05, ^**^p < 0.01,^***^p < 0.001, ^****^p < 0.0001, compared to CON group; ^#^*p* < 0.05, ^##^*p* < 0.01, ^###^*p* < 0.001, ^####^*p* < 0.0001, compared to DSS group.

Cytokine levels were quantified using enzyme-linked immunosorbent assay (ELISA) to assess the suppressive effects of BBR-EVO. Mice with DSS-induced colitis exhibited significantly elevated levels of IL-6, TNF-α, and IL-1β (Figure 4). Treatment with BBR and EVO effectively reduced the production of these cytokines in colitic mice, with a more pronounced reduction observed when BBR and EVO were combined.

### BBR-EVO inhibited the oxidative state

**Figure 5:**
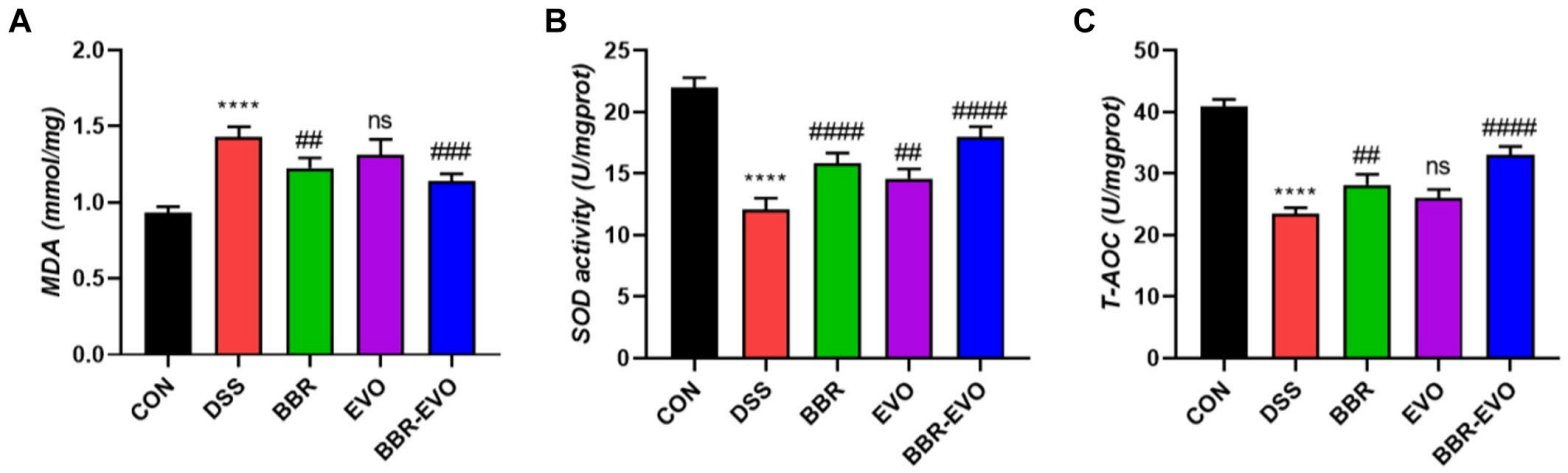
Effects of the active components of Yulian decoction on oxidant and antioxidant indices in liver homogenates of colitis mice, *n* = 5. **(A)** MDA. **(B)** SOD. **(C)** T-AOC. Data are presented by mean ± SD; ^*^p < 0.05, ^**^p < 0.01, ^***^p < 0.001, ^****^p < 0.0001, compared to CON group; ^#^*p* < 0.05, ^##^*p* < 0.01, ^###^*p* < 0.001, ^####^*p* < 0.0001, compared to DSS group.

At the same time, representative oxidative stress factors were also detected (Figure 5). The findings revealed that the DSS group’s MDA content was significantly higher than that of the CON group (*p* < 0.0001) and that the activities of SOD and T-AOC were reduced to varied degrees (*p* < 0.01). SOD and T-AOC levels considerably increased (*p* < 0.01) and MDA levels dramatically decreased (*p* < 0.01) under the administration of BBR and BBR-EVO, whereas MDA and T-AOC levels in the colon of mice treated with EVO alone did not significantly alter. Therefore, BBR-EVO may have the ability to inhibit inflammatory response and enhance antioxidant defense to inhibit DSS injury.

**Figure 6:**
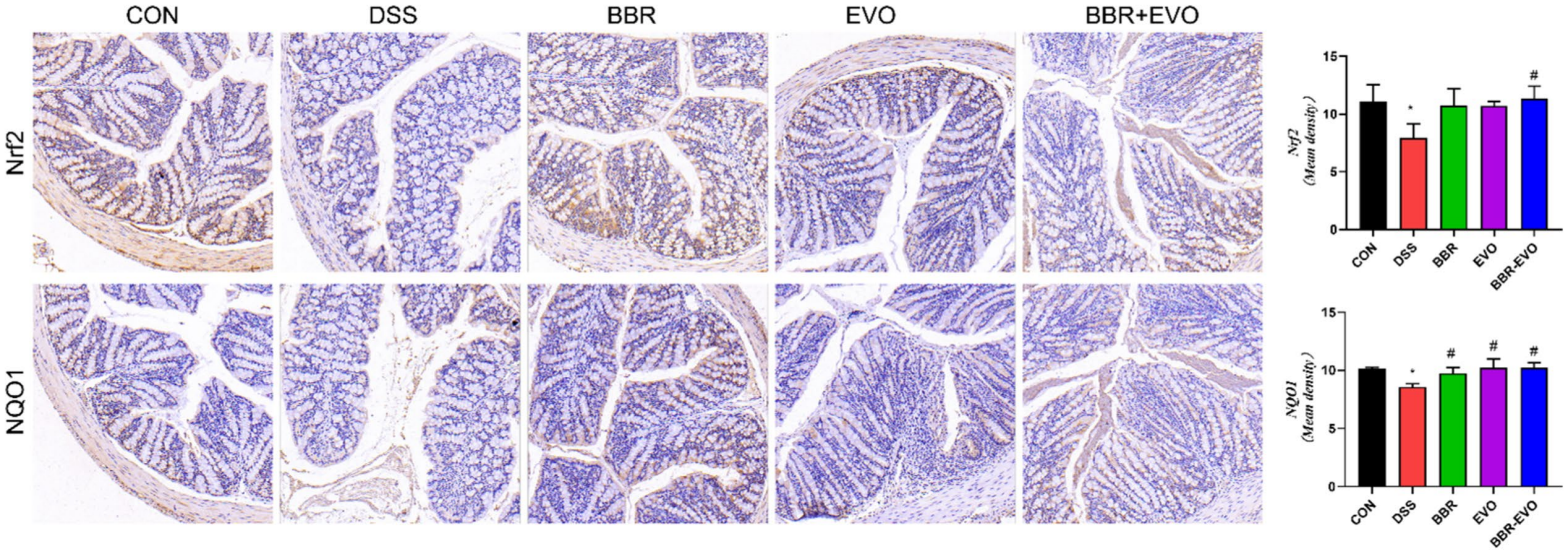
Effects of the main active components of Yulian decoction on the expression of antioxidant proteins nuclear factor erythroid 2-related factor 2 (Nrf2) and NAD(P)H quinone dehydrogenase 1 (NQO1) in the colon tissues of DSS-induced colitis mice, as determined by immunohistochemistry method. Scale bar = 50 μm.

The IHC staining findings indicated that the expression levels of nuclear factor erythroid 2-related factor 2 (Nrf2) and NQO1 in the DSS group’s colon tissue were lower than in the CON group, indicating that the antioxidant signaling pathway in the DSS group was suppressed (Figure 6). This is consistent with the antioxidant parameter test findings.

### BBR-EVO enhanced the tight junction proteins expressions of colon tissue in DSS-induced colitis mice

**Figure 7:**
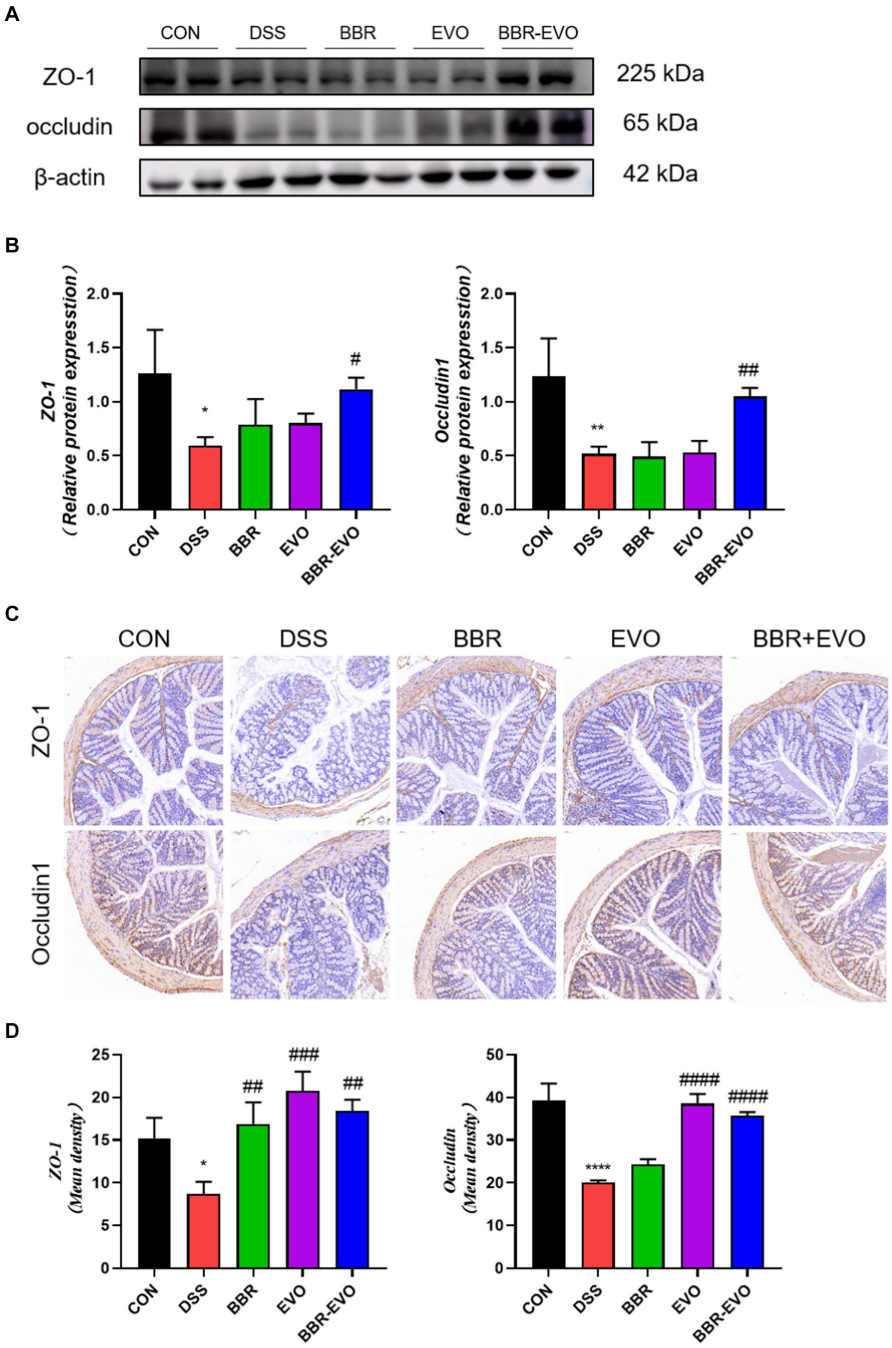
Effects of the active components of Yulian decoction on tight junction function in the colon tissues of colitis mice. **(A,B)** Protein expression of ZO-1 and occludin in colon tissues. **(C,D)** Immunohistochemical analysis results of ZO-1 and occludin. Scale bar = 50 μm. Data are presented by mean ± SD; ^*^p < 0.05, ^**^p < 0.01,^***^p < 0.001, ^****^p < 0.0001, compared to CON group; ^#^*p* < 0.05, ^##^*p* < 0.01, ^###^*p* < 0.001, ^####^*p* < 0.0001, compared to DSS group.

Intestinal barrier function is mainly attributed to intercellular tight junction proteins (ZO-1 and occludin) in colon tissue. As a result, we used Western blot and IHC labeling to examine the levels of ZO-1 and occludin expression in mice colon tissues (Figure 7). As predicted, the expression of ZO-1 and occludin was considerably lower in the DSS group than in the control group (*p* < 0.05). However, preventing BBR-EVO significantly reduced the reduction in ZO-1 and occludin expression caused by DSS (*p* < 0.05).

### Effects of BBR-EVO on the intestinal flora abundance of DSS-induced colitis in mice

**Figure 8:**
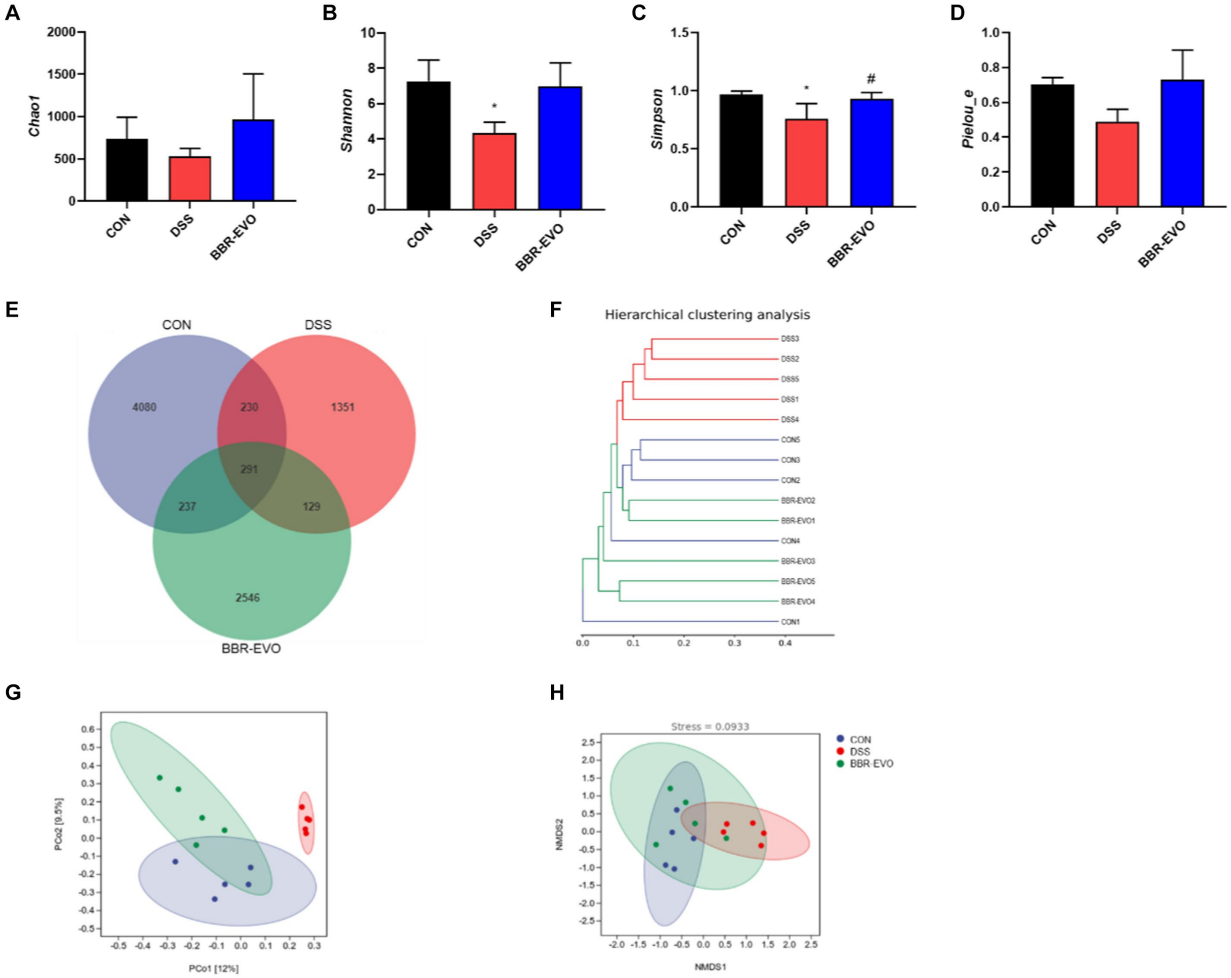
BBR-EVO regulates the abundance and composition of intestinal microbiota of DSS-induced colitis in mice, *n* = 5. **(A–D)** Indexes of Chao1, Shannon, Simpson and Pielou_e. **(E)** Venn diagram. **(F)** Hierarchical clustering analysis based on unweighted_unifrac distance matrices. **(G)** PCoA plot based on jaccard distance matrices. **(H)** NMDS analysis based on weighted_unifrac distance matrices. Data are presented by mean ± SD; ^*^p  < 0.05, ^**^p < 0.01,^***^p < 0.001, ^****^p < 0.0001, compared to CON group; ^#^*p* < 0.05, ^##^*p* < 0.01, ^###^*p* < 0.001, ^####^*p* < 0.0001, compared to DSS group.

We examined the gut microbiome structure to verify the impact of BBR-EVO on it. To confirm the effect of BBR-EVO on the intestinal microbiome, we analyzed the composition of the intestinal microbiome. α-diversity analysis showed that the Shannon and Simpson indices of mice were significantly decreased under DSS influence. However, BBR-EVO intervention significantly increased the Simpson index related to community richness and increased the diversity of microbial colonies to some extent. Whereas the community diversity determined by the Chao1 and Pielou_e indices did not change significantly (Figures 8A–D). The results of the Venn diagram showed that the numbers of different ASVs/OTUs between the BBR-EVO group and the CON group were both higher than those of the DSS group (Figure 8E). We used PCoA analysis, NMDS analysis, and sample hierarchical clustering analysis to measure β-diversity. The results showed that BBR-EVO was significantly distinct from the DSS group (Figures 8F–H). This indicates that BBR-EVO supplementation can significantly regulate the DSS-induced changes in the composition of the intestinal microbial structure.

**Figure 9:**
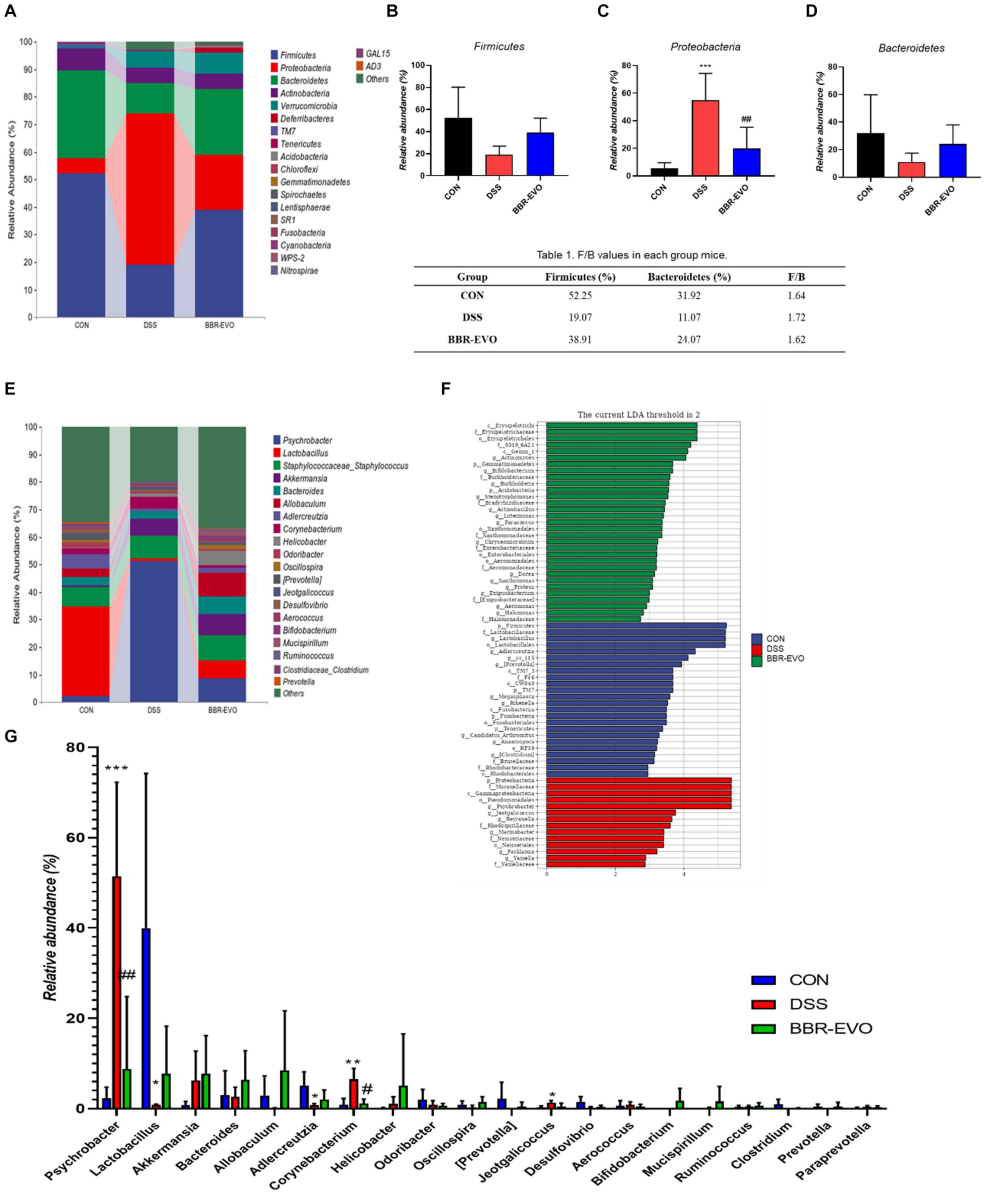
BBR-EVO changes the composition of intestinal microbiota in different taxa levels, *n* = 5. **(A–D)** Relative abundance of gut microbiota in Phylum level, Firmicutes abundances, Proteobacteria abundances and Bacteroidetes abundances. **(E)** Relative abundance of gut microbiota in genus level. **(F)** LEfSe analysis. **(G)** Relative abundance of specific genera. Data are presented by mean ± SD; ^*^p < 0.05, ^**^p < 0.01, ^***^p < 0.001, ^****^p < 0.0001, compared to CON group; ^#^*p* < 0.05, ^##^*p* < 0.01, ^###^*p* < 0.001, ^####^*p* < 0.0001, compared to DSS group.

The differences in gut microbiota composition across groups were further analyzed at the phylum and genus levels (Figure 9). After DSS treatment, mice exhibited a substantial increase in the relative abundance of *Proteobacteria* (*p* < 0.05), while the abundance of *Firmicutes* and *Bacteroidetes* decreased, and the ratio of *Firmicutes* to *Bacteroidetes* (F/B) increased slightly (*p* > 0.05) (Figures 9A–D; Table 1). The genus-level composition of the top 20 abundant taxa is depicted in Figure 9E. According to genera composition and LEfSe analysis, finding revealed considerable variations and development in the gut microbiome via BBR-EVO administration (Figures 9F,G). Compared to the CON group, the abundance of *Psychrobacter* and *Corynebacterium*, two important bacterial genera, was markedly higher in the DSS group, whereas the abundance of *Lactobacillus* was reduced. Following BBR-EVO administration, the abundance of *Psychrobacter* (*p* < 0.01) and *Corynebacterium* (*p* < 0.05) were significantly decreased, and the abundance of *Lactobacillus* was improved, although not significantly (*p* > 0.05). In addition, after DSS induction, the richness of *Adlercreutzia* decreased (*p* < 0.05) and that of *Jeotgalicoccus* increased (*p* < 0.05), which were slightly reversed by BBR-EVO (Figure 9G). In summary, whilst BBR-EVO does not re-establish normobiosis within the gastrointestinal microbiome subsequent to DSS perturbation, the data indicate that it may confer modulatory effects on the compositional variability and biodiversity of the gut microbiota. These alterations may contribute to an amelioration of the DSS-induced dysbiosis, suggesting a potential therapeutic role for BBR-EVO in the management of microbiota-mediated gastrointestinal pathologies.

### BBR-EVO alleviated liver damage in mice with DSS-induced colitis

**Figure 10:**
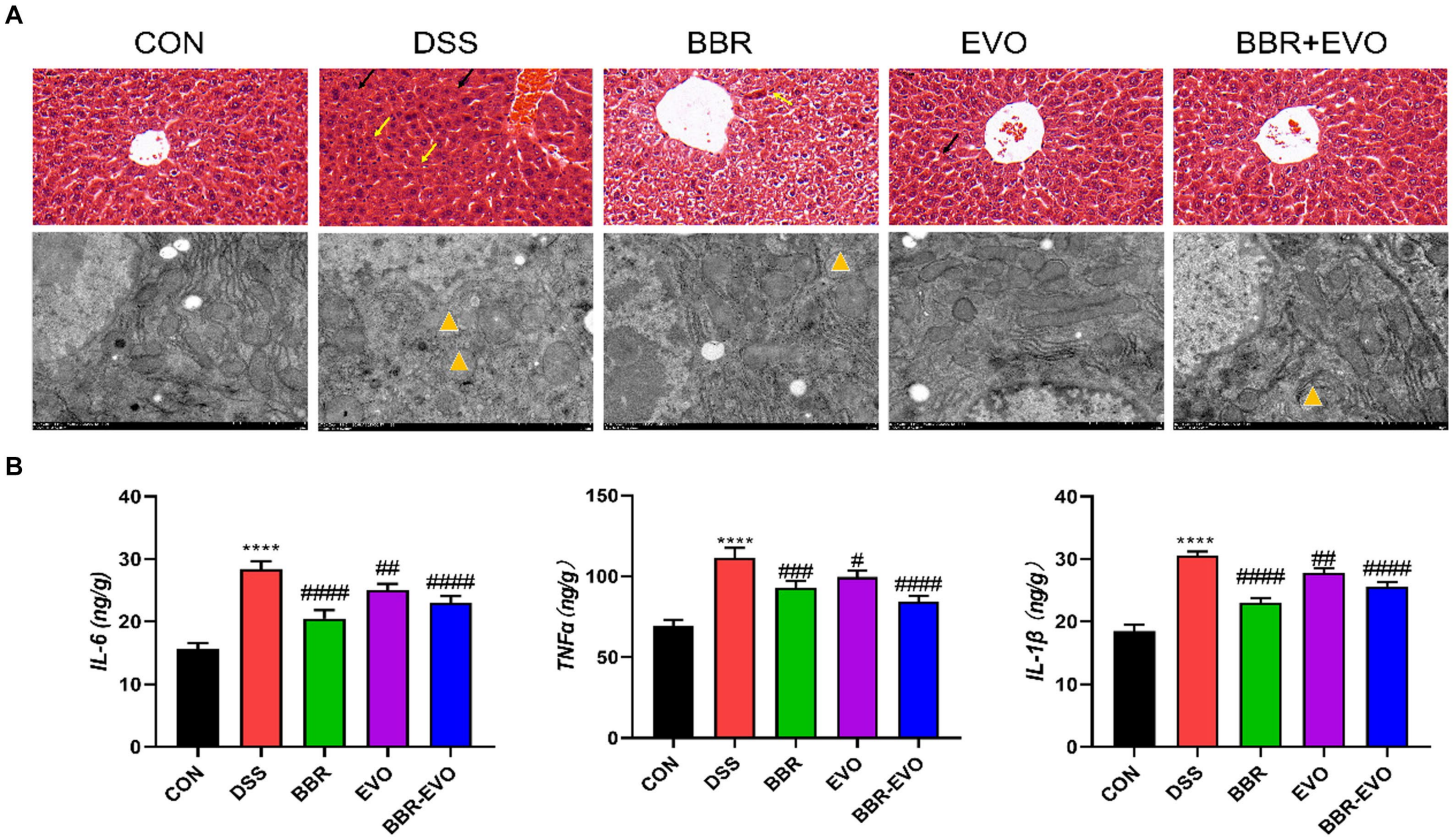
Effects of the active components of Yulian decoction on liver damage in colitis mice. **(A)** H&E staining (50 μm) and electron microscopy (2 μm). Black arrows indicate bleeding; Yellow arrows indicate inflammatory infiltrate; Yellow triangles show that mitochondria are swollen and vacuolated. **(B)** IL-6, TNF-α, IL-1β levels, *n* = 5. Data are presented by mean ± SD; ^*^p < 0.05, ^**^p < 0.01, ^***^p < 0.001, ^****^p < 0.0001, compared to CON group; ^#^*p* < 0.05, ^##^*p* < 0.01, ^###^*p* < 0.001, ^####^*p* < 0.0001, compared to DSS group.

This experiment evaluated the attenuating effect of BBR-EVO on colitis-related liver injury. H&E staining and electron microscopy revealed that DSS-induced liver histological damage was characterized by immune cell infiltration, even local hemorrhage, a reduction in mitochondrial count, cellular edema, and mitochondrial vacuolization (Figure 10A). Post-treatment assessment with BBR, EVO and BBR-EVO revealed a diminution in both liver structural and ultrastructural damage in mice. Furthermore, ELISA analyses demonstrated that, relative to the CON group, hepatic concentrations of pro-inflammatory cytokines—IL-6, TNF-α, and IL-1β—were significantly elevated in the DSS group. Contrastingly, these inflammatory markers were substantially reduced in the liver tissues of the mice subjected to the therapeutic interventions (Figure 10B). This suggests that BBR-EVO might exert hepatoprotective effects in the context of DSS-induced colitis.

### BBR-EVO reduced DSS-treated mice from liver oxidative stress

**Figure 11:**
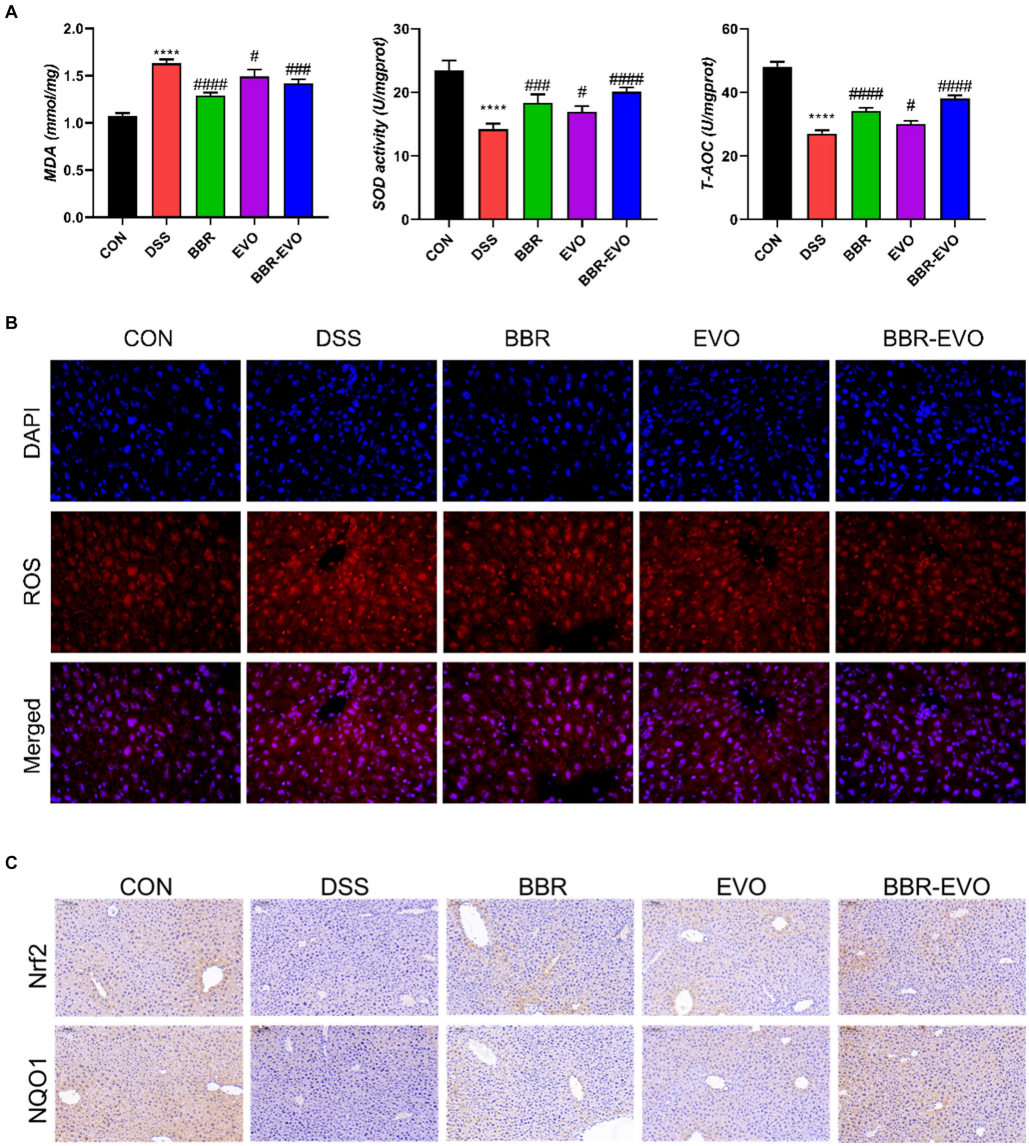
Effects of the active components of Yulian decoction on liver oxidative damage in colitis mice, *n* = 5. **(A)** MDA, SOD and T-AOC levels. **(B)** Fluorescence of ROS (400×). **(C)** Immunohistochemical analysis results of Nrf2, NQO1. Scale bar = 50 μm. Data are presented by mean ± SD; ^*^p < 0.05, ^**^p < 0.01, ^***^p < 0.001, ^****^p < 0.0001, compared to CON group; ^#^*p* < 0.05, ^##^*p* < 0.01, ^###^*p* < 0.001, ^####^*p* < 0.0001, compared to DSS group.

When compared to colitis mice, liver T-AOC and SOD levels were considerably higher following BBR, EVO, and BBR-EVO intervention, while liver MDA levels were significantly lower (Figure 11A). Similarly, as compared to the DSS group, the frozen section data indicated a drop in ROS levels in the liver, indicating a reduction in oxidative stress (Figure 11B). Furthermore, liver IHC staining revealed that colitis caused oxidative stress in the liver, which BBR-EVO may significantly relieve by stimulating the expression of the Nrf2/NQO1 protein (Figure 11C).

## Discussion

In our study, we explored the therapeutic potential of BBR-EVO, a principal component derived from the traditional Yulian decoction, on a model of colitis elicited by DSS administration. Our findings indicate that BBR-EVO not only attenuates the damage to the colon induced by DSS but also promotes the recovery of intestinal barrier functions and reduces permeability issues. The compound demonstrated a capacity to suppress inflammatory responses in the colon and to effect positive changes in the gut microbiota, which in turn contributed to a decrease in associated liver damage and oxidative stress. This interaction appears to be mediated by the gut-liver axis. Our research methodology was informed by a pioneering concept known as “Preparations Quality Markers” (p-Marker) (Du et al., 2021; Li T. et al., 2021; Ran et al., 2022), which was developed by our team. This innovative approach provides a systematic framework for the identification and quantification of bioactive substances within complex herbal formulations. By applying this strategy, we were able to establish an effective ratio of BBR to EVO, ensuring the therapeutic efficacy of BBR-EVO in our colitis model.

Intestinal health is frequently linked to a wide range of disorders, including cardiovascular disease, gastrointestinal disease, and even cancer. Interestingly, intestinal inflammation can cause an imbalance in the body’s immune, resulting in increased inflammation in other organs such as the liver, breast, and brain (Hu et al., 2023; Zhao et al., 2023; Zou et al., 2023). In recent years, intestinal flora has been considered as a key factor in ulcerative colitis. According to the α-diversity and β-diversity index, DSS reduced the diversity and richness of intestinal flora in fecal samples (Agus et al., 2018). Consistent with previous studies, the relative abundance of gut microbiota was shown at the phylum level. The DSS group in this study also showed ecological imbalance of gut microbiota, including a significant decrease in the abundance of *Bacteroides* and *Firmicutes*, a slight increase in the ratio of *Firmicutes*/*Bacteroides* (F/B), and an increase in the abundance of *Proteobacteria* (Shin et al., 2015), accompanied by the loss of microbial diversity. Our study showed that BBR-EVO could reverse the F/B ratio in DSS-treated mice and decreasing the abundance of *Proteobacteria.* An increase in the F/B ratio is considered a sign of intestinal flora disturbance (Hou et al., 2022). *Proteobacteria* is thought to be the primary pathogenic bacterium, which is capable of producing endotoxins (Vester-Andersen et al., 2019; Wang et al., 2020; Zhu et al., 2020). The inflammatory reaction to DSS might be connected to the rise in *Proteobacteria* abundance (Wang et al., 2020). *Psychrobacter* was prominent in the digestive tracts of DSS-treated mice and belonged to the *Proteobacteria* genus. Although limited is known about the clinical significance of *Psychrobacter*, several *Psychrobacter* species have been known to cause conjunctivitis, peritonitis, endocarditis, infant meningitis, arthritis, surgical wound infection, and bacteremia, mainly in immunocompromised patients (García-López et al., 2014), such as the gut of immunodeficiency virus infection (Qing et al., 2019). We found a significant change in the abundance of *Corynebacterium* in the gut microbiota. *Corynebacterium* is considered to be one of the microorganisms associated with bacteremia (Yamamuro et al., 2021). Another study found that cadmium exposure induced *Corynebacterium* to move from the intestines to the blood (Liu et al., 2023). *Jeotgalicoccus* is a member of the *Staphylococcaceae* family known to be associated with colitis (Li H. et al., 2021), which was further confirmed by this study. In addition, *Staphylococcus aureus*, which frequently leads to inflammation, is especially prevalent in people with digestive system problems (Ikeuchi et al., 2006). In addition, BBR-EVO pretreatment reduced the levels of IL-1β, IL-6 and TNF-α in colon tissue. *Adlercreutzia* has been shown to be a probiotic or positively associated with human/animal health (Galipeau et al., 2021). After DSS treatment, the abundance of *Adlercreutzia* in the intestine decreased significantly (Galipeau et al., 2021; Song et al., 2023). In this study, BBR-EVO could reverse the disturbance of DSS on intestinal flora, significantly reduce the richness of *Psychrobacter* and *Corynebacterium*, and slightly adjust the richness of *Jeotgalicoccus* and *Adlercreutzia*, and confirm that the possible mechanism may be related to the improvement of intestinal function by balancing the micro-environment of the gut microbiota.

IBD is a gastrointestinal dysfunction caused by an immune response. Furthermore, intestinal barrier failure of the context of disease in the organism, accompanied by the aggravation of inflammation, can cause disturbed immune homeostasis in gut and allow harmful exogenous compounds to enter the body and induce liver damage (Cash et al., 2006; Hu et al., 2020; Pabst et al., 2023; Wang et al., 2023; Yang et al., 2023). It has been reported that alkaloids have unique advantages in the treatment of ulcerative colitis due to their multiple targets and high safety (Zhang et al., 2018; Li Q. et al., 2021; Li et al., 2022). This study showed disordered colon tissue with intestinal villus destruction and intercellular tight junction destruction in DSS group mice. The expression levels of Occludin and ZO-1 protein in colon tissue of DSS group mice were significantly decreased, indicating that the tight junction of intestinal mucosal barrier was destroyed after the establishment of ulcerative colitis model, which was consistent with previous research results (Cui et al., 2021). After BBR-EVO intervention, the intestinal tissue structure damage, inflammation and intestinal oxidative stress in mice were alleviated, which was speculated to be related to the repair of intestinal barrier by BBR-EVO. According to our hypothesis, the disruption of intestinal flora, particularly the growth of *Proteobacteria*, has been proven to be one of the origins of endotoxin (Wang et al., 2020). Flora metabolites or certain flora metabolites reach the liver via the intestinal barrier and cause liver injury (Fang et al., 2018). By measuring the inflammatory indexes of the liver, we proved that the liver tissue of DSS model mice had inflammatory cell infiltration, increased inflammatory factors, and destroyed liver tissue structure. In addition, in the preventive intervention of BBR-EVO, the degree of liver damage in mice was reduced. Our study also demonstrated that BBR-EVO might protect the liver in DSS-induced colitis by activating the Nrf2-ARE-NQO1 pathway and improving mitochondrial function. These findings align with previous research on Nrf2’s role in liver protection (Liu et al., 2024). Increased Nrf2 and NQO1 expression following BBR-EVO treatment is consistent with berberine’s known ability to activate Nrf2 signaling (Li et al., 2023), ultimately increasing the activity of antioxidant enzymes. BBR-EVO also mitigated mitochondrial damage, significant given the role of mitochondrial dysfunction in liver diseases (Qin et al., 2019; Yang et al., 2024). The interplay between Nrf2 activation, antioxidant capacity, and mitochondrial function creates a protective against liver inflammation and oxidative damage.

Although our study confirmed that BBR-EVO has a protective effect on ulcerative colitis mice by regulating the gut-liver axis related to gut microbiota to reduce oxidative stress and inflammatory response in the liver and intestine, there are still some limitations in this study. The relationship between gut bacteria, gut and liver is complex and dynamic. Whether the increase and decrease of related microbiota are related to the degree of liver injury has not been directly proved by more experiments. Therefore, we need to continue to study *in vitro* and *in vivo* experiments in the next step.

## Data Availability

The datasets presented in this study can be found in online repositories. This data can be found here: https://www.ncbi.nlm.nih.gov/, accession number PRJNA1150401.

## Footnotes

^1^ https://www.genescloud.cn
